# Tooth surface loss associated with unshelled sunflower seed consumption: a scoping review

**DOI:** 10.3389/froh.2026.1889799

**Published:** 2026-09-25

**Authors:** Suresh Kandagal Veerabhadrappa, Srinivas Sulugodu Ramachandra, Seema Yadav, Pooja Adtani, Thantrira Porntaveetus

**Affiliations:** 1 Department of Oral Diagnostic Sciences, Faculty of Dentistry, SEGi University, Kota Damansara, Petaling Jaya, Selangor, Malaysia; 2 Dental Research Cell, Dr. D. Y. Patil Dental College & Hospital, Dr. D. Y. Patil Vidyapeeth (Deemed to be University), Pune, India; 3 Department of Restorative Dentistry, College of Dental Medicine, University of Sharjah, Sharjah, United Arab Emirates; 4 Department of Periodontics, Faculty of Dentistry, SEGi University, Kota Damansara, Petaling Jaya, Selangor, Malaysia; 5 Department of Basic Medical and Dental Sciences, College of Dentistry, Gulf Medical University, Ajman, United Arab Emirates; 6 Center of Excellence in Precision Medicine and Digital Health, Chulalongkorn University Implant and Esthetic Center, Faculty of Dentistry, Chulalongkorn University, Bangkok, Thailand; 7 Clinic of General, Special Care and Geriatric Dentistry, Center for Dental Medicine, University of Zurich, Zurich, Switzerland

**Keywords:** abrasion, incisal notching, professional education, sunflower seeds, tooth surface loss, tooth wear

## Abstract

**Objectives:**

The consumption of unshelled sunflower seeds (USS) is a common, culturally embedded dietary habit in many parts of the world. However, the dental findings associated with this practice have not been comprehensively synthesised. This scoping review aimed to map the available evidence on the reported dental findings associated with USS consumption and to identify gaps in existing literature.

**Materials and methods:**

Following the PRISMA Extension for Scoping Reviews (PRISMA-ScR) guidelines, a comprehensive search of PubMed, MEDLINE, Scopus, Embase, Web of Science, and Google Scholar was conducted for studies published from inception to August 2026. Eligibility was based on the Population, Concept, and Context(PCC) framework. English-language peer-reviewed cross-sectional studies, case series, case reports, and narrative reviews reporting dental findings associated with USS consumption were included and descriptively synthesized.

**Results:**

Eight articles (two cross-sectional studies, three case reports, two case series, and one narrative review with a previously unpublished case) met the inclusion criteria. Tooth surface loss (TSL), presenting as characteristic incisal notching of the anterior teeth was the primary finding associated among the included studies. Infrequently reported findings included incisal fractures, dentinal hypersensitivity, gingival recession, widening of the periodontal ligament space, and localized inflammatory peri-implant disease associated with food impaction. Temporomandibular joint (TMJ) symptoms and masticatory muscle tenderness were reported only in a single case series.

**Conclusion:**

USS consumption may be associated with TSL, commonly presenting as characteristic incisal notching of the anterior teeth. Reported periodontal and TMJ findings can be considered as exploratory observations. Although TSL has been reported in association with USS consumption, the current evidence is insufficient to establish a causal relationship. Further epidemiological and longitudinal studies are needed to clarify these associations and determine the prevalence, progression, and clinical significance of the reported oral findings.

## 1 Introduction

The consumption of abrasive dietary seeds, particularly unshelled sunflower seeds, is common in several parts of the world (1). Unshelled sunflower seeds (USS) are inexpensive and widely available in dried, roasted, or salted forms, although they are occasionally consumed in their natural form (2, 3). Derived from the plant *Helianthus annuus* L., these seeds are an integral part of the dietary and cultural practices of certain ethnic communities in Southeast Asia and are also commonly consumed in China, particularly among the Chinese population (4). This culturally embedded practice is often enjoyed during social gatherings, as a leisure snack, or as a means of stress relief, largely because of the recognised nutritional value and perceived health benefits of sunflower seeds (2, 4–6). Sunflower seeds are known for their antioxidant properties and contain healthy unsaturated fats, protein, fibre, and various trace elements, making them a functional food that can help prevent hypercholesterolemia, arterial hypertension, obesity, and cancer (7, 8). They also exhibit anti-inflammatory, skin-protective, analgesic, and antibacterial effects (9, 10).

During consumption, the sunflower seed is placed with the pericarp intact, between the maxillary and mandibular anterior teeth, where the shell is cracked by applying sufficient biting force to separate it from the kernel. The shell is subsequently discarded and the kernel is consumed (11) (Figure 1). This habit requires repeated protrusive and biting movements of the mandible, exposing the anterior teeth to repetitive mechanical loading. Over time, repeated shell cracking may contribute to irreversible tooth surface loss (TSL) (11).

**Figure 1:**
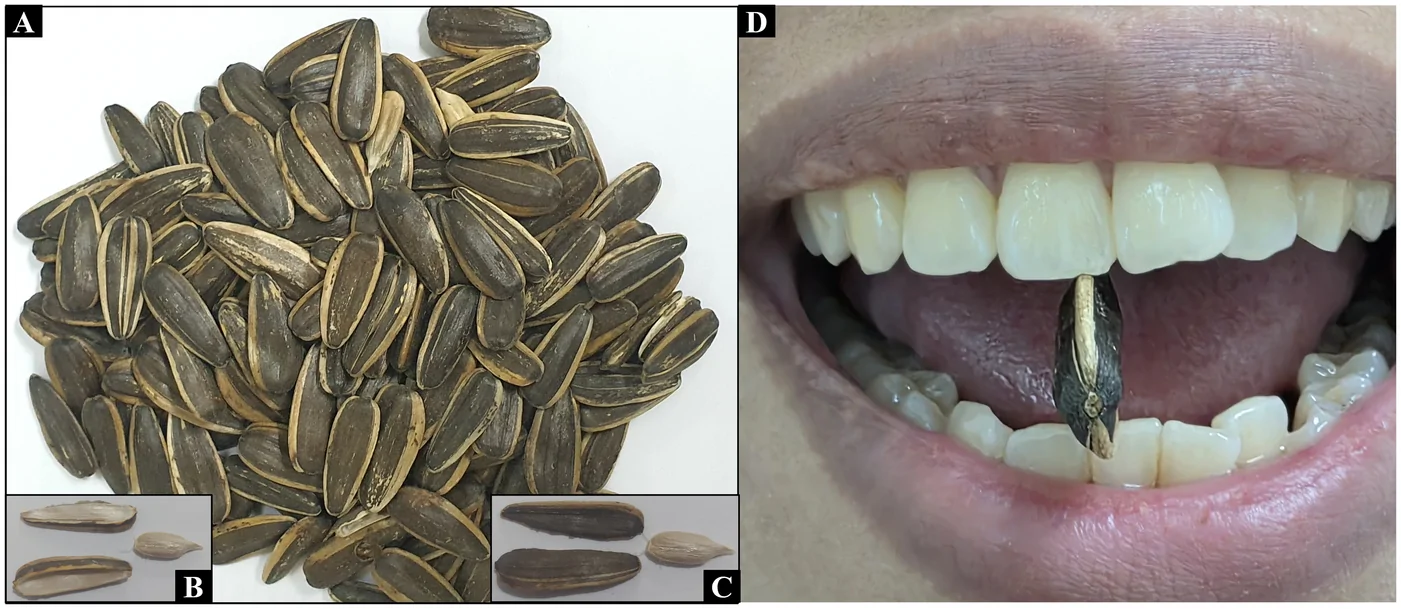
demonstrates the pattern of placement of USS between maxillary and mandibular incisors with their shell being intact. Image of USS **(A)**, shells of the sunflower seeds and the kernel of the sunflower seeds **(B and C)**, the facial view of the patient with the USS placed in the notches (Figure 1D). **(D)** is an unpublished clinical photograph obtained from a patient seen by the authors. Written informed consent was obtained from the patient. No patient identifying details are presented.

TSL refers to the irreversible loss of dental hard tissues resulting from non-carious processes, including attrition, abrasion, erosion, and abfraction (12). Attrition is the loss of dental hard tissues caused by tooth-to-tooth contact during mastication. Erosion refers to the loss of dental hard tissues due to chemical processes, whereas abfraction describes cervical lesions that may develop because of circumflexural occlusal forces. In contrast, abrasion is the pathological wear of tooth structure caused by mechanical factors other than tooth-to-tooth contact (13). The TSL associated with USS consumption is most consistent with abrasion because repeated shell cracking subjects the incisal edges of the anterior teeth to continuous mechanical wear.

Dietary seeds, such as pumpkin and watermelon seeds (14–16), have also been reported to contribute to TSL because of their abrasive potential. However, USS are hypothesised to have greater abrasive potential due to the repeated cracking of their shells between the teeth. Previous reports have documented incisal edge damage associated with harmful oral habits, including chewing abrasive tobacco products (17), as well as regular USS consumption (11, 12, 16). In addition, impacted sunflower shells have been reported as a cause for localized inflammatory peri-implant disease (18). However, the available evidence remains limited and is insufficient to establish a causal relationship between USS consumption and the reported dental findings. To date, no comprehensive evidence synthesis has examined the dental health implications of USS consumption. Therefore, this scoping review was undertaken to map and synthesise the existing evidence on the dental findings associated with USS consumption.

## 2 Materials and methods

### 2.1 Reporting guidelines

The framework proposed by Arksey and O’Malley was utilized while undertaking this scoping review (19). This approach facilitates a comprehensive mapping of the available literature on the topic. The review was conducted in accordance with the Preferred Reporting Items for Systematic Review and Meta Analyses Extension for Scoping Reviews (PRISMA-ScR) guidelines (20). This scoping review was not prospectively registered, and no review protocol was registered on an external platform.

### 2.2 Framework and review question

The review question, eligibility criteria, and search strategy were developed according to the Population, Concept, and Context (PCC) framework. **Population (P):** Individuals with a history of USS consumption; **Concept (C):** Dental findings reported in association with USS consumption; **Context (C):** Clinical, community, and research settings without restrictions on geographic location, age, sex, or ethnicity. Within the context of this review, an exposure to USS consumption can be defined as placement of the USS with its pericarp intact between the maxillary and mandibular anterior teeth, and the shell being cracked by application of sufficient biting force to separate it from the kernel.

### 2.3 Review question

The review aimed to address the following question “What dental findings have been reported in the literature among individuals with a history of USS consumption?”

### 2.4 Search strategy

A comprehensive literature search was independently undertaken by two authors (SKV and SRS) in the PubMed, MEDLINE, Scopus, Embase, and Web of Science databases for studies published from inception to August 2026 that reported dental findings associated with USS consumption. Additionally, potential articles from the grey literature were searched in Google Scholar from inception to August 2026 using the Publish or Perish software (21).

The literature search was developed using two concept groups: (1) exposure terms related to USS consumption (“sunflower seeds”, “shelled sunflower seeds”, “natural sunflower seeds”, “dried roasted seeds”, “sunflower seed chewing”, “seed chewing habits”, “oral habits”, and “seeds”) and (2) outcome terms related to tooth surface loss (“incisor notching”, “incisal edge abrasion”, “tooth surface loss”, “tooth craze lines”, “tooth wear”, “dental abrasion”, “mechanical tooth wear”, and “attrition”). Within each concept group, search terms were combined using the Boolean operator OR, and the two concept groups were combined using AND.

The complete database-specific search strategies, including the fully parenthesised Boolean search strings as executed, database platform, search date, field tags, publication and language limits, and database-specific search syntax, are provided in Supplementary Table S1. The Google Scholar search was conducted using the Publish or Perish software (21) using a single, simplified combined keyword string because of the search functionality and character limitations, as Google Scholar search allows only 256 characters (Supplementary Table S1).

### 2.5 Inclusion and exclusion criteria

Case reports, case series, cross-sectional studies, longitudinal observational studies, and narrative reviews (in case containing any original data) published in English describing clinical findings associated with USS consumption were included. Only studies involving human participants published from inception to 21st August 2026 were included. Studies were included when the reported dental findings were described in relation to USS consumption. If the dental findings could not be specifically attributed to USS consumption because of the presence of a confounding factor, such studies were excluded. Animal studies, cadaveric studies, *in vitro* investigations, conference abstracts, and publications that did not report dental findings associated with USS consumption and studies examining TSL or other dental conditions unrelated to USS consumption were excluded. Non-English publications were not considered.

### 2.6 Study selection

All retrieved records from different databases were exported to EndNote X5 (Clarivate Analytics), and duplicates were removed using the “Find Duplicates” tool (Library). Subsequently, two reviewers (SKV and SRS) independently screened titles and abstracts for relevance. Full-text articles were assessed for eligibility based on the inclusion criteria. Disagreements were resolved through discussion, and if needed following consultation with a third reviewer (SY). Reference lists of included articles were screened to identify additional relevant publications.

### 2.7 Data extraction, collating, summarizing and result reporting

To ensure standardization, data were charted using a structured data extraction form adapted for scoping reviews (22–24). Data extraction was guided by the PCC framework and information included: author, year, country, type of article, study aim, study design, population/sample size, exposure type, directness of attribution, diagnostic methods, dental findings, outcome domain and limitations (Table 1). Two reviewers (SKV and SRS) independently extracted the data, with discrepancies resolved through discussion. For the case series (25), published by members of the current review team, eligibility assessment and data extraction were performed independently by a co-author (PA) who was not involved in the original case series.

**Table 1:** Characteristics of included studies and summary of reported dental findings associated with unshelled sunflower seed consumption.

Authors, Year, Country and Type of Article	Aims of the Study	Study Design	Population and sample size	Exposure Type	Directness of attribution and reported competing causes	Diagnostics Methods	Major Findings	Outcome Domain	Limitations
Al-Habahbeh et al., 2011, (16) Jordan, Cross-sectional study.	To evaluate the prevalence and factors affecting TSL.	Cross-sectional study involving a questionnaire, interviews and clinical examination.	Patients with TSL associated with consumption of dried roasted seeds. Total sample: 141 participants. Dried-roasted seed consumers: 80 (56.7%). USS-specific exposure: Not reported. Participants with the relevant TSL findings: Not reported	Mixed-seed exposure.	Mixed seed and competing causes reported.	Clinical examination of TSL and its assessment using the Smith and Knight index. Direct interviews with patients, and a questionnaire to assess the factors affecting TSL.	Incisal notching was observed predominantly on the incisal edges of the central incisors and canines.	Dental hard tissues.	USS were evaluated together with other dried roasted seeds. Therefore, the specific contribution of USS consumption was not determined.
Rusu Olaru et al., 2019 (28) Romania, Cross-sectional study.	To assess the etiological factors involved in the occurrence of non-carious cervical lesions.	Cross-sectional study involving clinical examination and questionnaire to evaluate TSL.	Patients with non-carious TSL. Total sample: 21 patients with TSL. USS exposure: 2 participants. Participants with incisal notching: 2 (9.52%).	USS-specific.	Direct. No competing causes reported.	The clinical examination of TSL was conducted, and its severity was evaluated using the Smith and Knight index. Subsequently, a questionnaire was used to assess the etiological factors contributing to TSL.	Two patients (9.52%) presented with notching of the incisal edges of the maxillary right central incisors.	Dental hard tissues.	USS consumption was reported alongside other potential causes of TSL, limiting attribution of findings specifically to USS seed consumption. The study examined a small sample of 21 patients between the ages of 20 and 72 who presented with TSL.
Bidra A.S. 2014, (18) United States, Case report.	To report localized inflammatory peri-implant disease/foreign-body complication caused by lodging of the sunflower seed shell around the peri-implant sulcus of a dental implant.	Case report.	Patient with implant-supported over denture.	USS-specific	Direct. No competing causes reported.	Clinical examination History of USS consumption. Intraoral radiographs were taken for confirmation.	A sunflower seed shell was found impacted within the peri-implant sulcus, accompanied by erythema, suppuration, and peri-implant inflammation.	Localized inflammatory peri-implant disease with food impaction.	The effects of USS on other dental hard tissues were not reported. Exposure type, frequency and duration were not reported.
Rath et al., 2017, (11) Malaysia, Case report.	To report a case of incisal notching attributed to long-term USS consumption.	Case report	One female and one male patient with a history of USS consumption. Two cases	USS-specific.	Direct. No competing causes reported.	History and clinical examination of dental hard tissues to evaluate TSL.	Prominent notching extending approximately 2 mm into the enamel of the maxillary and mandibular incisors. A positive family history of USS consumption was reported.	Dental hard tissues.	The authors did not mention the effects of USS on the periodontium.
Warreth et al., 2020, (12) Saudi Arabia, Narrative Review with original data.	To provide an overview of the classification and management of TSL.	Narrative Review.	Patient consuming USS. One case	USS-specific.	Direct. No competing causes reported.	Clinical examination attributing USS consumption to incisal notching in maxillary and mandibular central incisors.	Incisal notching affecting the maxillary and mandibular central incisors were reported.	Dental hard tissues.	Frequency and duration of the USS consumption and its effect on the hard and soft tissues were not reported. This is a narrative review and contains an unpublished case of TSL due to USS consumption. So only the original case is considered and not the entire review article.
Yüce M et al., 2025 (26) Turkey, Case report	To report localised incisal notching in a male patient caused due to USS consumption.	Case report.	A 38-year-old male patient with a history of USS consumption. One case	USS-specific	Direct. No competing causes reported.	Clinical examination of the patient with a history of USS consumption. Intraoral radiographs were taken for confirmation of periodontal ligament widening.	Localized notching extending 1–2 mm into the enamel was observed on teeth 11 and 41, resulting in an aesthetic concern.	Dental hard tissues and periodontium	The authors did not report the effects of USS consumption on other oral tissues, the TMJ, or the muscles of mastication. Furthermore, habits frequency and duration were not reported.
Vanishree et al., 2025, (27). Malaysia, Case series.	To report a series of cases of TSL associated with USS consumption.	Case series.	Eight patients (4 males and 4 females) with a history of USS consumption. Seven patients were Malaysian and one patient was of Iranian nationality. Eight cases	USS-specific	Direct. No competing causes reported.	Detailed dietary, social and occupational history was obtained. Clinical examination assessed incisal edge notching, with photographic documentation of affected teeth.	Twenty-two anterior teeth showed incisal edge notching. The maxillary right central incisor was the most frequently affected tooth. The authors highlighted dietary history as important for diagnosis.	Dental hard tissues.	Exposure characteristics, such as frequency, duration, quantity, and cumulative habit history were not reported. Diagnosis was based on clinical history and examination without standardised diagnostic criteria.
Veerabhadrappa SK et al., 2026, (25) Malaysia, Case series.	To report the oral health implications of USS chewing.	Case series.	Eleven cases of Chinese ethnic origin.	USS-specific.	Direct. No competing causes reported.	The clinical case history and examination of eleven patients included an assessment of the teeth, periodontium, and temporomandibular joint (including the masticatory muscles) to evaluate the effects of USS consumption on these structures.	V-shaped and U-shaped incisal notches were observed, with deeper lesions (1–2 mm) observed in frequent consumers. Additional findings included dentinal hypersensitivity, incisal edge fractures, tooth discoloration, and craze lines. Widening of the periodontal ligament space, interdental bone loss, and temporomandibular symptoms were associated with USS consumption in a small number of cases. A positive family history of USS consumption was noted.	Dental hard tissues, periodontium and TMJ.	The case series lacked comprehensive details regarding habit history, type and quantity of USS consumed, diagnostic procedures, and the management protocols adopted for each case.

TMJ: Temporomandibular Joint; TSL: Tooth Surface Loss; USS: Unshelled Sunflower Seeds.

USS-specific consumption refers to the process of consuming the kernel of unshelled sunflower seeds. During this process, the USS with its pericarp intact is placed between the maxillary and mandibular incisors with the application of force to break open the shell. Subsequently, the kernel is consumed.

Mixed-seed exposure refers to studies in which studies report patients consuming USS and other dried roasted seeds, making it difficult to determine the specific contribution of USS to TSL.

Methodological characteristics of included studies were descriptively summarised to map the nature, design, and scope of the available evidence. Given the heterogeneity of study designs and outcome reporting, findings were synthesised narratively and formal critical appraisal was not performed.

## 3 Results

### 3.1 Identified records and included studies

The initial search identified a total of 651 articles (PubMed = 69; MEDLINE = 72; Scopus = 139; Embase = 53; Web of Science = 118; and Google Scholar = 200). After removing 239 duplicates, 412 articles were screened based on titles and abstracts. Twenty-two (*n* = 21) full-text articles were assessed for eligibility and following full-text review by two independent reviewers (SKV and SRS), eight articles were included in the review. Among the eight articles, seven studies were from primary sources, and one was a narrative review with a previously unpublished case presenting TSL due to USS consumption. The selection process with identified, screened, eligible and included articles are illustrated in Figure 2. The screenshots of the search in each of the databases are provided in Supplementary Figures S1-S6, Supplementary File. The articles included in the review are listed in Supplementary Table S2 of the Supplementary File. The articles excluded after full-text review with reasons are listed in Supplementary Table S3 of the Supplementary File.

**Figure 2:**
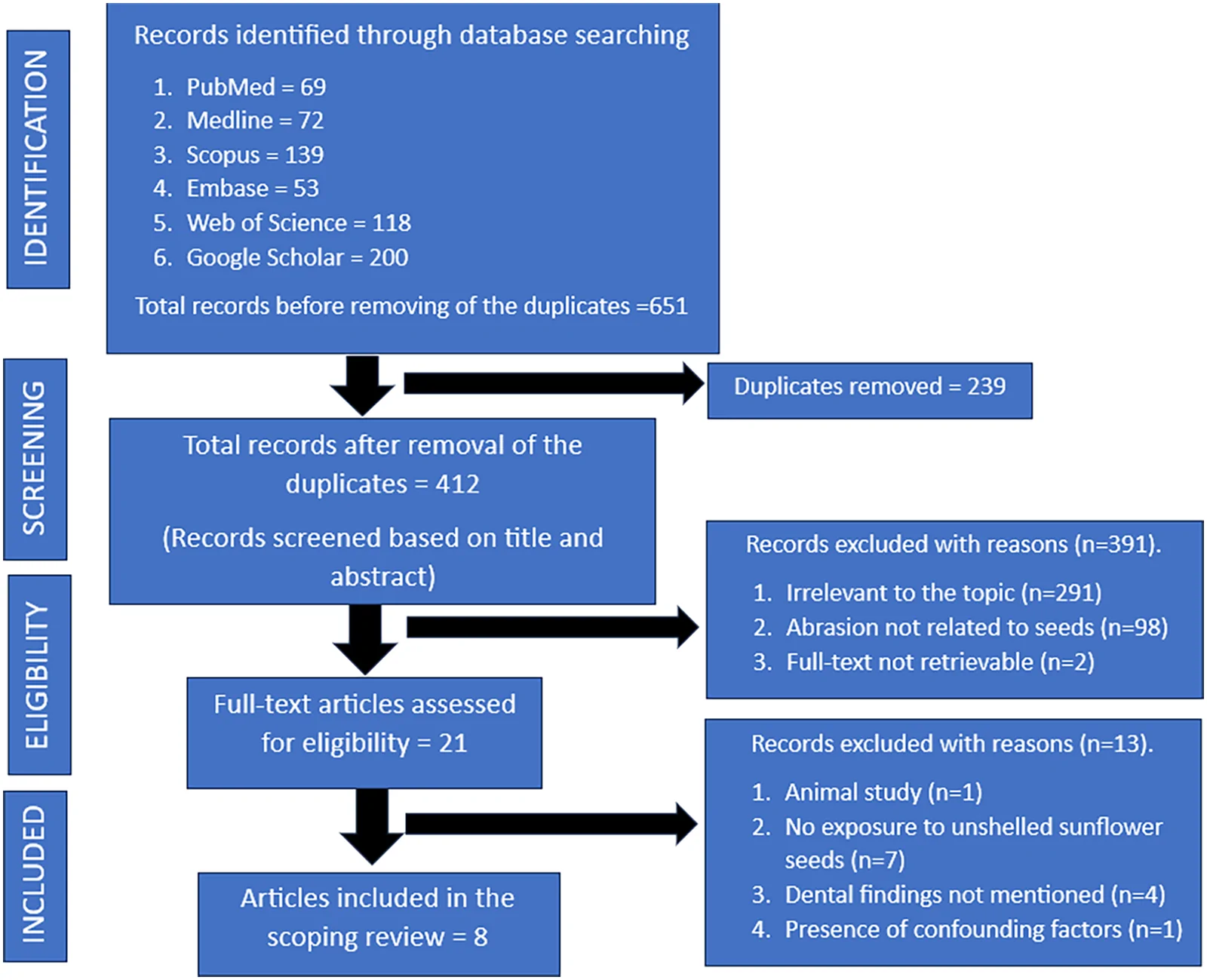
PRISMA-ScR flow diagram of study selection process.

The included studies reflected the PCC framework by reporting dental findings among individuals with USS consumption. The evidence comprised of three case reports, one each from the United States (18), Turkey (26), and Malaysia (11), two case series from Malaysia (25, 27), two cross-sectional studies, one each from Jordan (16) and Romania (28), and a narrative review (containing a unpublished case) from Saudi Arabia (12).

### 3.2 Characteristics of included studies

Most studies investigated individuals with a history of USS consumption; however, one cross-sectional study evaluated sunflower seeds together with other dried roasted seeds, limiting attribution of the findings specifically to USS consumption (16). Reporting of exposure characteristics was inconsistent across studies. Information on the type of seed, frequency and duration of consumption, quantity consumed, cumulative exposure, and shell-cracking technique were frequently incomplete or not reported. Where available, the duration of the habit ranged from several months to more than three decades (11, 25). Familial patterns and culturally embedded consumption practices were also described in selected case reports and the case series (11, 25, 26).

### 3.3 Dental findings associated with USS consumption

The available evidence primarily described dental hard-tissue changes, with TSL presenting as characteristic incisal notching being the most consistently reported finding. Incisal notching was reported in two cross-sectional studies (16, 28), two case reports (11, 26), two case series (25, 27), and one narrative review (12). Variations in lesion morphology included V-shaped and U-shaped notches affecting the maxillary and mandibular incisors. Deeper lesions extending into dentin, dentinal hypersensitivity, incisal fractures, enamel craze lines, and tooth discoloration were reported in one case series (25).

Incidental findings included periodontal findings (25), localized inflammatory peri-implant disease due to food impaction (18), and temporomandibular joint (TMJ) symptoms (25). Periodontal findings, including gingival recession, gingival bleeding, widening of the periodontal ligament space, and interdental bone loss, were reported in one case series (25). A single case report described localized inflammatory peri-implant disease associated with impaction of a sunflower seed shell within the peri-implant sulcus (18). TMJ symptoms, including joint sounds and masticatory muscle tenderness, were also reported in one case series involving long-term USS consumers (25). Given the limited evidence, periodontal, TMJ findings and localized inflammatory peri-implant diseases associated with seed shell impaction should be regarded as exploratory observations rather than established manifestations of USS consumption. An evidence map of the dental and oral findings, hypothesised mechanisms, and management suggestions associated with USS consumption is presented in Figure 3.

**Figure 3:**
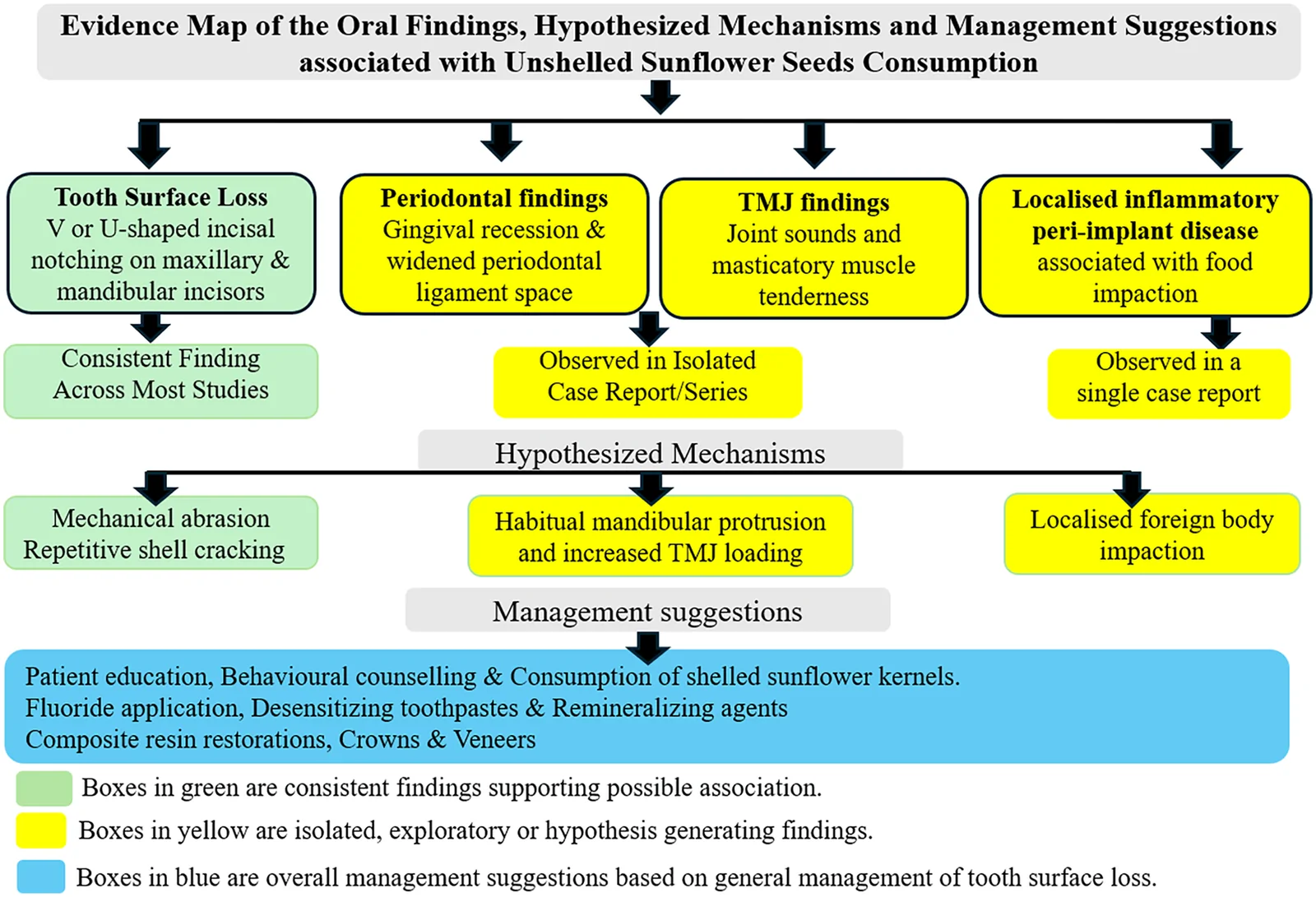
Evidence map of the oral findings, hypothesised mechanisms, and management suggestions associated with USS consumption. V- or U-shaped incisal notching was consistently reported across most studies, supporting a possible association (green boxes). Periodontal findings, TMJ findings, and localized inflammatory peri-implant disease associated with seed shell impaction were reported only in isolated cases and should be considered exploratory or hypothesis-generating (yellow boxes). Proposed mechanisms for these findings are also presented. Proposed management approaches are extrapolated from general tooth-wear literature or expert-informed clinical considerations.

## 4 Discussion

This scoping review mapped the available evidence on the dental findings associated with USS consumption. The current evidence base is of low-level and predominantly descriptive, consisting mainly of case reports, case series, cross-sectional studies, and a narrative review. Most publications originated from Malaysia (11, 25, 27), and Middle Eastern countries (12, 16), where USS consumption appears to be a culturally embedded habit. Although this practice is also common in other regions, including parts of East Asia, published clinical evidence remains scarce. This geographic concentration highlights the need for epidemiological and longitudinal studies in populations where USS consumption is prevalent. This article employed a scoping review methodology rather than a systematic review because the available evidence was limited and heterogeneous. The primary aim was to map and characterise the existing evidence and identify knowledge gaps requiring further investigation. Accordingly, this review was not intended to establish causality, estimate risk, or provide evidence-based clinical recommendations.

TSL, most commonly presenting as characteristic incisal notching of the anterior teeth, was the most consistently reported finding across the included studies (11, 12, 16, 25–28). However, the low level of available evidence does not allow the relationship between exposure and disease severity to be fully characterised, as details regarding the type of seed, frequency and duration of consumption, cumulative exposure, and shell-cracking technique were inconsistently reported. In addition, one cross-sectional study evaluated USS together with other dried roasted seeds, limiting the ability to attribute the reported findings exclusively to USS consumption (16).

Drying and roasting of USS, particularly when prepared with salt and citric acid may increase the abrasive potential of the seed shell (16). The incisal notching reported across the included studies is plausibly explained by repetitive mechanical contact between the USS and the anterior teeth during shell cracking. However, this proposed mechanism has not been investigated in the included studies. Additionally, possible synergistic effects of citric acid coatings and mechanical wear have also been suggested, but this remains hypothetical, as none of the included studies evaluated the potential erosive contribution of citric acid or other seed coatings. Therefore, the mechanisms underlying the development and progression of TSL remain uncertain and represent an important area for future research.

The available evidence suggests that TSL associated with USS consumption may be identified by its characteristic pattern of V or U-shaped incisal notching, particularly when accompanied by a relevant dietary history (25, 28). In several reports, these lesions were identified during routine dental examinations, as patients often did not seek treatment because the habit was culturally accepted or influenced by socioeconomic factors (11, 12, 25).

Information on the severity of incisal notching in relation to the frequency and duration of USS consumption was derived primarily from individual case reports (11) and case series (25, 27). Although dentinal hypersensitivity was reported in severe cases, the available evidence is insufficient to establish a dose response relationship between USS consumption and the severity or progression of TSL because of the descriptive nature of the studies and the absence of longitudinal data.

The narrative review by Warreth et al. (12) was included because it reported one previously unpublished case of TSL associated with USS consumption. Excluding this case would have resulted in the omission of original clinical data on a topic with very limited published evidence, thereby justifying its inclusion. No other articles or cases discussed in the review by Warreth et al. (12) were included in this scoping review. Consequently, duplicate reporting and double counting of clinical findings were avoided throughout the evidence synthesis. Among the excluded articles, one was a case report describing incisal notching associated with USS consumption (29). However, the primary focus of the report was to report on the adverse effects associated with methamphetamine use rather than the dental effects of USS consumption. Since several confounding factors including drug-induced bruxism, xerostomia and severe non-carious and carious TSL are associated with methamphetamine use (29), the evidence for a USS-specific incisal notching becomes indirect. Therefore, it was decided to exclude this case report (29).

Although TSL associated with USS consumption is generally considered to be abrasion-related, it should be viewed within the broader framework of non-carious TSL. The characteristic incisal notching is most likely related to repetitive mechanical wear between the sunflower seed shell and the incisal edges. However, inconsistent reporting of exposure characteristics across the included studies limited comparisons between studies and precluded assessment of potential dose–response relationships. Consequently, it was not possible to determine whether the frequency, duration, or cumulative exposure to USS consumption influenced the severity or progression of the reported dental findings.

Beyond TSL, a small number of studies reported additional oral findings, including gingival recession, widening of the periodontal ligament space, and alveolar bone changes (25, 26). These observations were based on isolated case reports and a single case series and therefore should be interpreted cautiously. A single case report also described localized inflammatory peri-implant disease due to food impaction resulting from impaction of a sunflower seed shell within the peri-implant sulcus, representing a localized foreign-body complication rather than evidence of a broader periodontal effect of USS consumption (18).

TMJ symptoms were reported in a single case series involving long-term USS consumers (25). Although repetitive shell cracking may increase functional loading of the masticatory system, this mechanism has not been directly investigated in individuals with USS consumption. Previous studies have associated repetitive oral parafunctional habits and temporomandibular disorders, suggesting a plausible mechanism for increased functional loading of the TMJ and masticatory muscles (30–32). However, these studies did not investigate USS consumption specifically.

The diagnosis of TSL associated with USS consumption is primarily based on characteristic incisal notching (Figures 4A,B) in conjunction with a relevant dietary history (11, 25–27). Careful evaluation of oral habits and medical history based on general tooth-wear and clinical management literature may help clinicians differentiate these lesions from other causes of TSL (Table 2) (33–37). TSL severity may be assessed using established indices such as the Smith and Knight Index (38) and the Basic Erosive Wear Examination (39).

**Figure 4:**
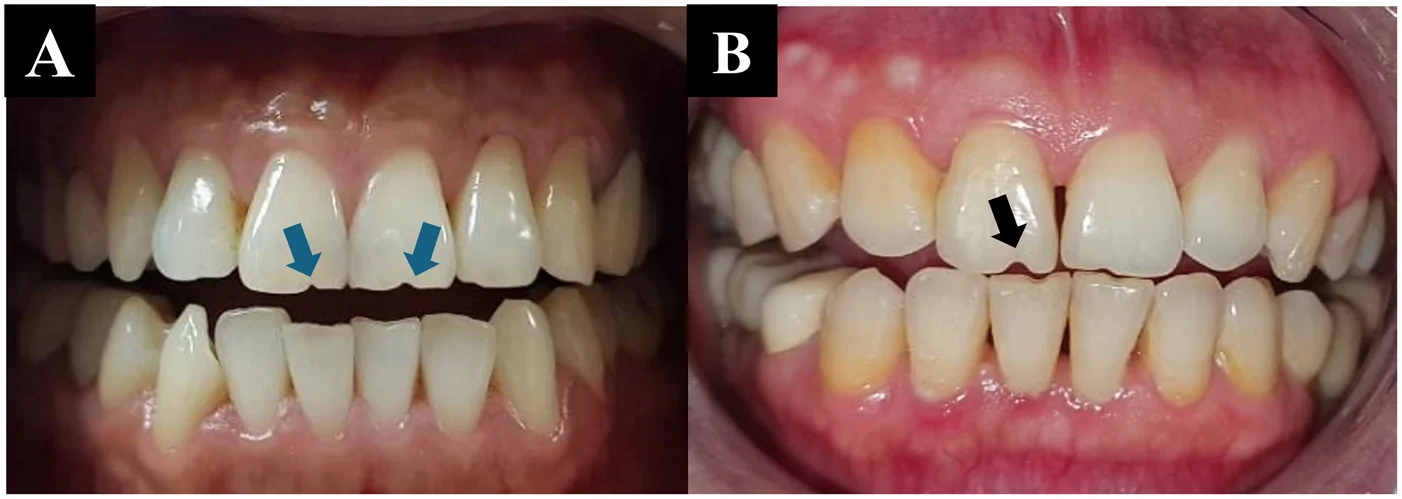
Clinical photographs showing **(A)** mild incisal notching (blue arrows) and **(B)** advanced incisal notching (black arrows) associated with USS consumption. Figures 4A,B are unpublished clinical materials obtained from patients seen by the authors. Written informed consent was obtained from the patient. No patient identifying details are presented.

**Table 2:** provides an overview of clinical signs, habit history and extraoral examination findings (33–37).

Assessment	Findings
**Clinical Signs**	• V- or U-shaped notching of the incisal edges.
	• Presence of crack lines and fractures on the incisal edges of the incisors.
**Habit History**	• A positive history of USS consumption is a key diagnostic feature, including details on the type (natural, dried roasted, salted) and frequency of consumption.
	• Information on the consumption of other seeds, such as watermelon and pumpkin seeds, should also be recorded.
	• Other deleterious oral habits should be ruled out, including pen-chewing, nail-biting, pipe-smoking, and occupation-related habits such as holding screws or pins between the teeth.
	• Review of medical history is essential to rule out systemic conditions like eating disorders and gastroesophageal reflux disease.
**Extraoral Examination**	• Examination of the TMJ and associated musculature for clicking, crepitus, mandibular deviation, limited jaw opening, and muscle tenderness. Currently, there is limited evidence to support the association of USS consumption with TMJ findings from isolated case reports/series.

TMJ: Temporomandibular Joint; USS: Unshelled Sunflower Seeds.

Management approaches described in the literature include educating patients about the irreversible nature of TSL and providing counselling on habit modification, particularly within culturally sensitive contexts (40). Community awareness initiatives have also been proposed to encourage safer practices, such as consuming dehulled sunflower kernels instead of cracking the shells intraorally (26). However, no intervention studies have evaluated the effectiveness of these behavioural strategies. Consequently, the management approaches outlined in Table 3 are based primarily on the general management approaches for TSL based on the severity (41–43) rather than evidence specific to USS-associated lesions. Furthermore, as no studies have evaluated treatment outcomes in individuals with USS-associated TSL, the effectiveness of these management approaches remains uncertain.

**Table 3:** provides the general management approaches for tooth surface loss based on the severity (41–43).

S.no	Severity of TSL	Proposed management approaches were extrapolated from general tooth-wear literature or expert-informed clinical considerations.
1	TSL involving only enamel.	• Behavioural counselling and recommendation s to consume dehulled sunflower kernels rather than cracking the shells intraorally.
		• Preventive treatments include professional fluoride applications, desensitizing toothpastes, and remineralizing agents such as casein phosphopeptide-amorphous calcium phosphate toothpastes.
2	TSL involving both enamel and dentin.	• Behavioural counselling and preventive approaches (above mentioned).
		• Restorative treatment using composite resin restorations can be considered.
3	TSL with additional involvement of the periodontium.	• Behavioural counselling, preventive approaches and restorative treatment (above mentioned).
		• Intraoral peri-apical radiographs may be indicated to detect the presence of periodontal involvement.
		• Interdisciplinary treatment involving periodontal therapies, crowns and veneers can be considered.

TSL: Tooth Surface Loss; USS: Unshelled Sunflower Seeds. No studies specifically evaluating the management of USS-associated TSL were identified in this review. Therefore, the effectiveness of these management approaches for USS-associated TSL remains uncertain.

The findings of this review should be interpreted in light of the limited and predominantly descriptive nature of the available evidence. Although the included studies reported TSL presenting as incisal notching, they were not designed to establish causality, incidence, progression, or dose–response relationships. In contrast, the association of periodontal changes, localized inflammatory peri-implant disease associated with seed shell impaction, and TMJ findings with USS consumption in isolated case reports should be regarded as preliminary, hypothesis-generating observations. In addition, several potential confounding factors, including bruxism, vomiting, dietary acid exposure, tobacco use, poor oral hygiene, gastro-oesophageal reflux, eating disorders and other recognised causes of TSL, may have contributed to the reported dental findings.

Several important research gaps were identified. Epidemiological studies are needed to determine the prevalence of USS consumption and its associated dental findings across different populations. Prospective cohort studies with appropriately matched comparison groups are required to establish causal relationships, while dose–response studies should evaluate whether the frequency, duration, and cumulative exposure to USS consumption is associated with the severity and progression of dental findings. Biomechanical investigations are also needed to quantify the forces generated during shell cracking and their effects on dental tissues.

Future studies should adopt standardized exposure definitions, including the type of seed, frequency and duration of consumption, cumulative exposure, and shell-cracking technique, together with validated diagnostic criteria for dental outcomes. Multicentre, cross-cultural epidemiological studies involving diverse populations are also needed to understand the prevalence and clinical characteristics of USS-associated oral findings across different geographical regions and cultural practices. Finally, studies evaluating preventive strategies, behavioural interventions, and the use of deshelled sunflower kernels as a safer alternative are required.

This review has several limitations. First, the available evidence was predominantly descriptive, limiting the generalizability of the findings. Second, the consumption of USS is specific to certain regions and is culturally embedded in specific ethnicities. Although grey literature was searched in Google Scholar, there could be articles which may have been missed due to exclusion of non-English articles. Lack of funding for translating possible non-English articles needs to be acknowledged as a limitation. Third, few studies evaluated sunflower seeds together with other dried roasted seeds, preventing attribution of the reported findings exclusively to USS consumption. Therefore, these findings should be interpreted with caution. Fourth, the periodontal, TMJ, and severity findings were reported in a single case series authored by members of the review team. Therefore, the association between USS consumption and these findings should be interpreted with caution. Finally, heterogeneity in study designs, populations, exposure reporting, and outcome assessment limited comparisons across studies.

## 5 Conclusion

The available evidence suggests a reported association between USS consumption and TSL, most commonly presenting as characteristic incisal notching of the anterior teeth. In contrast, periodontal changes, localized inflammatory peri-implant disease associated with seed shell impaction, and TMJ findings have been associated in only a small number of studies and should be regarded as exploratory observations. Although the current evidence is insufficient to establish a causal relationship, USS consumption should be considered a possible contributing exposure rather than an established risk factor for TSL.
