# Supplementary material for: Tooth surface loss associated with unshelled sunflower seed consumption: a scoping review
Source: Front Oral Health. 2026 Sep 25;7:1889799. doi: 10.3389/froh.2026.1889799 (PMC13650472; doi:10.3389/froh.2026.1889799)
Supplement: Supplementary file 1 [file Supplementaryfile1.pdf]

Supplementary File

Title: Tooth Surface Loss and Oral Findings Associated with Unshelled Sunflower Seed Consumption: A Scoping Review.

Supplementary Table S1: Detailed search strategy for each of the databases.		
**Databases**	**Search strategy**	**Results**
PubMed	**Search platform:** NCBI PubMed<br>**Search date:** 21st August 2026<br>**#1** ("Sunflower Seeds*" OR "Shelled Sunflower Seeds*" OR "Natural Sunflower Seeds*" OR "Dried Roasted Seeds*" OR "Sunflower Seed Chewing*" OR "Seed Chewing Habits*" OR "Oral Habits*" OR Seeds*)<br>**#2** ("Incisor Notching*" OR "Incisal Edge Abrasion*" OR "Tooth Surface Loss*" OR "Tooth Craze Lines*" OR "Tooth Wear*" OR "Dental Abrasion*" OR "Mechanical Tooth Wear*" OR Attrition*)<br>**#3** #1 AND #2<br>**Limits applied:**<br>• Publication date: Inception – 21st August 2026<br>• Language: English<br>**Note:** The PubMed search strategy is reported exactly as executed. During the search, apart from the results, a notice that eight quoted phrases were not found in the phrase index was obtained.	69
MEDLINE	**Search platform:** EBSCOhost<br>**Search date:** 21st August 2026<br>**Field searched:** EBSCOhost search fields (no field restriction applied)<br>**# 1** (“Sunflower Seeds*" or "Shelled Sunflower Seeds*" or "Natural Sunflower Seeds*" or "Dried Roasted Seeds*" or “Sunflower Seed Chewing*” or “Seed Chewing Habits*” or “Oral Habits*” or Seeds*)<br>**AND**<br>**# 2** (“Incisor Notching*” or "Incisal Edge Abrasion*" or "Tooth Surface Loss*" or “Tooth Craze Lines*” or “Tooth Wear*” or “Dental Abrasion*” or “Mechanical Tooth Wear*” or Attrition*)<br>**#1 AND # 2**<br>Limits applied:<br>• Publication date: Inception – 21st August 2026.	72

Scopus	**Search platform:** Elsevier Scopus<br>**Search date:** 21st August 2026<br>**# 1** TITLE-ABS-KEY ("Sunflower Seeds*" OR "Shelled Sunflower Seeds*" OR "Natural Sunflower Seeds*" OR "Dried Roasted Seeds*" OR "Sunflower Seed Chewing*" OR "Seed Chewing Habits*" OR "Oral Habits*" OR Seeds*)<br>**# 2** TITLE-ABS-KEY (“Incisor Notching*" OR "Incisal Edge Abrasion*" OR "Tooth Surface Loss*" OR "Tooth Craze Lines*" OR "Tooth Wear*" OR "Dental Abrasion*" OR "Mechanical Tooth Wear*" OR Attrition*)<br>**# 1 AND # 2**<br>Limits applied:<br>• Publication date: Inception- 21st August 2026<br>• Language: English	139
Embase	**Search platform:** Ovid Embase<br>**Search date:** 21st August 2026<br>**#1** ("Sunflower Seeds*" OR "Shelled Sunflower Seeds*" OR "Natural Sunflower Seeds*" OR "Dried Roasted Seeds*" OR "Sunflower Seed Chewing*" OR "Seed Chewing Habits*" OR "Oral Habits*" OR Seeds*)<br>**#2** Limit #1 to publication years 1980–2026<br>**#3** ("Incisor Notching*" OR "Incisal Edge Abrasion*" OR "Tooth Surface Loss*" OR "Tooth Craze Lines*" OR "Tooth Wear*" OR "Dental Abrasion*" OR "Mechanical Tooth Wear*" OR Attrition*)<br>**#4** Limit #3 to publication years **2000–2026**<br>**#5** #2 AND #4<br>**Limits applied:**<br>• Publication year: 1980–2026	53
Web of Science	**Search platform:** Web of Science<br>**Search date:** 21st August 2026<br>**#1** (TS= (“Sunflower Seeds*" or "Shelled Sunflower Seeds*" or "Natural Sunflower Seeds*" or "Dried Roasted Seeds*" or “Sunflower Seed Chewing*” or “Seed Chewing Habits*” or “Oral Habits*” or Seeds*))<br>**# 2** (TS = (“Incisor Notching*” or "Incisal Edge Abrasion*" or "Tooth Surface Loss*" or “Tooth Craze Lines*” or “Tooth Wear*” or “Dental Abrasion*” or “Mechanical Tooth Wear*” or Attrition*))<br>**#1 AND # 2**<br>**Limits applied:**<br>• Index date: Inception–21st August 2026.<br>Language: English	118
Google Scholar	**Search platform:** Google Scholar<br>**Search date:** 24th August 2026<br>**Combined keyword string:** Sunflower Seeds|Shelled Sunflower Seeds|Natural Sunflower Seeds|Seed Chewing Habits|Oral	200

	Habits|Incisor Notching|Tooth Surface Loss|Tooth Craze Lines|Tooth Wear|Dental Abrasion|Mechanical Tooth Wear|Attrition<br>**Limits applied:**<br>• Publication date: Inception–24th August 2026.<br>Language: English	

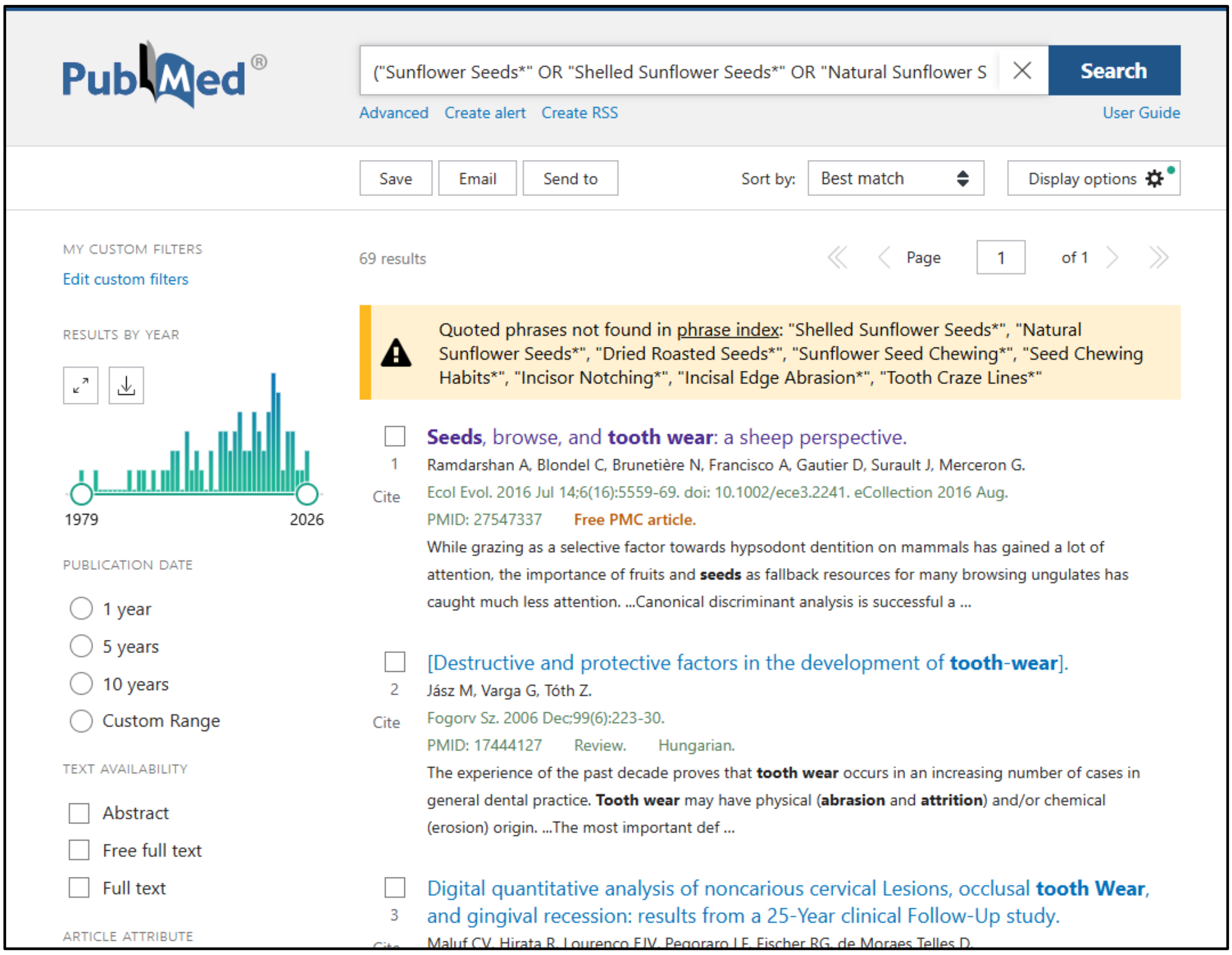

Supplementary Figure S1: Screenshot of results in PubMed

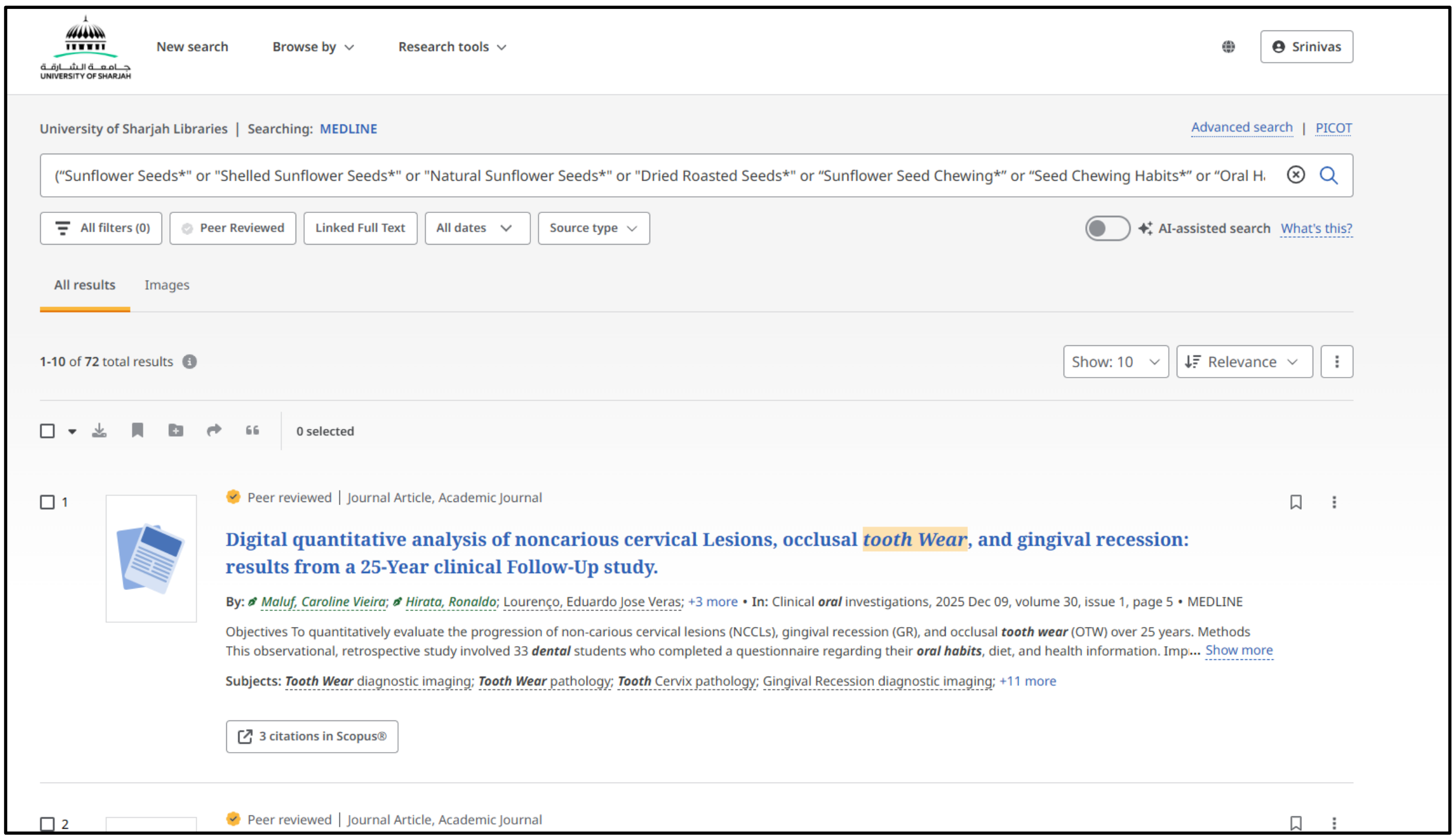

Supplementary Figure S2: Screenshot of search results in MEDLINE.

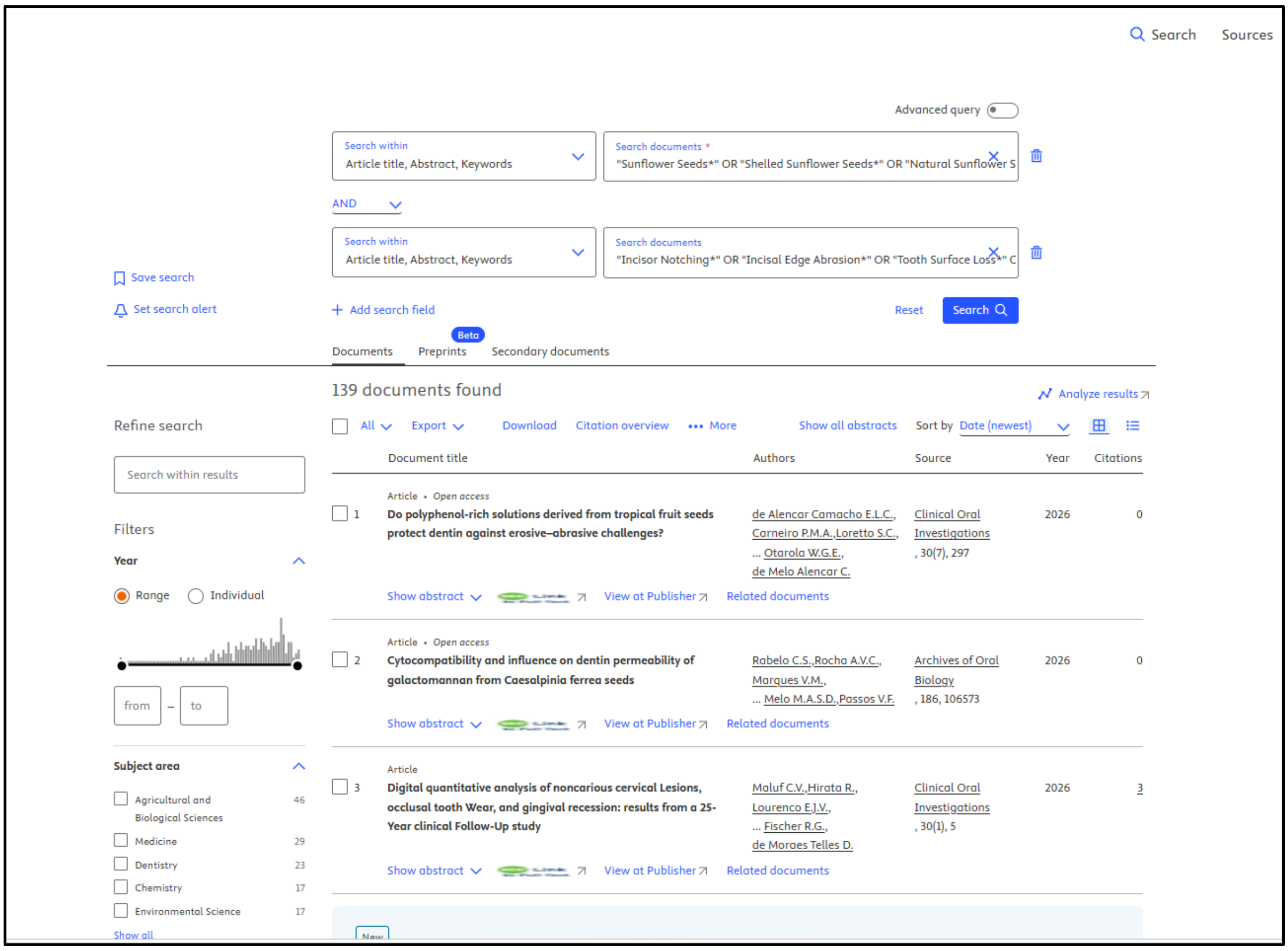

Supplementary Figure S3: Screenshot of search results in Scopus.

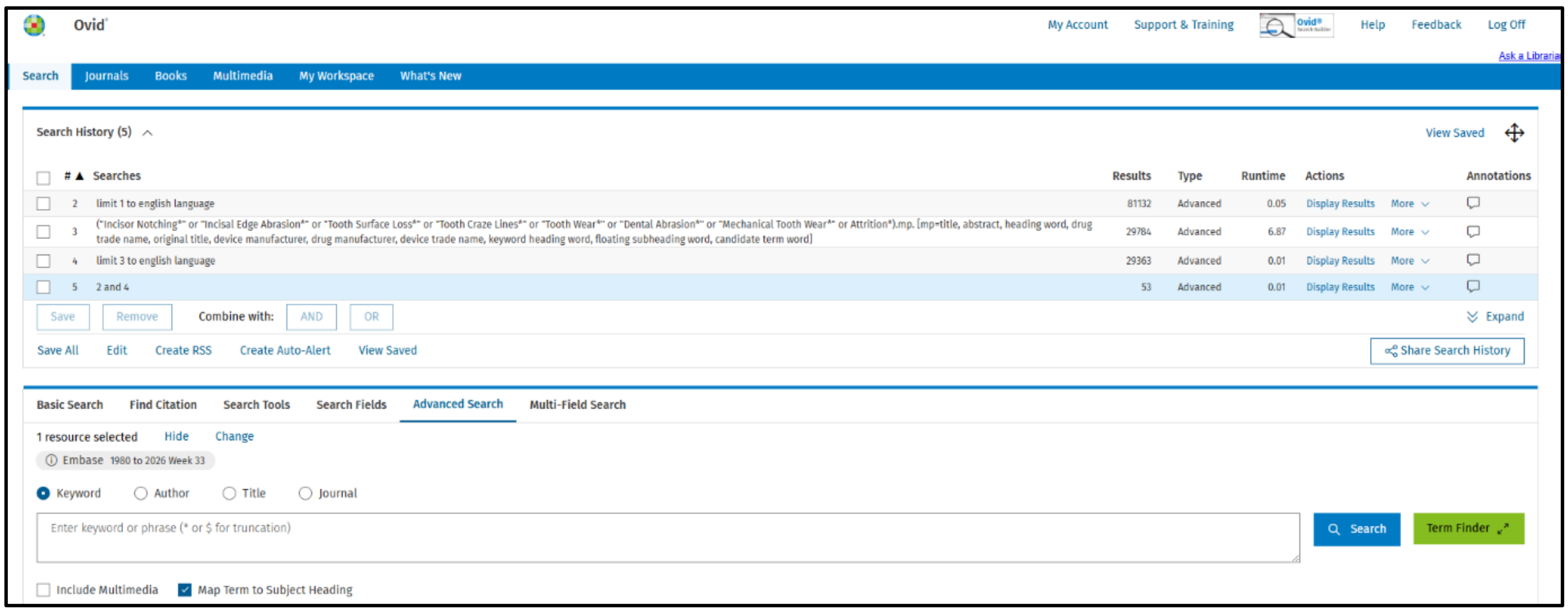

Supplementary Figure S4: Screenshot of search results in Embase

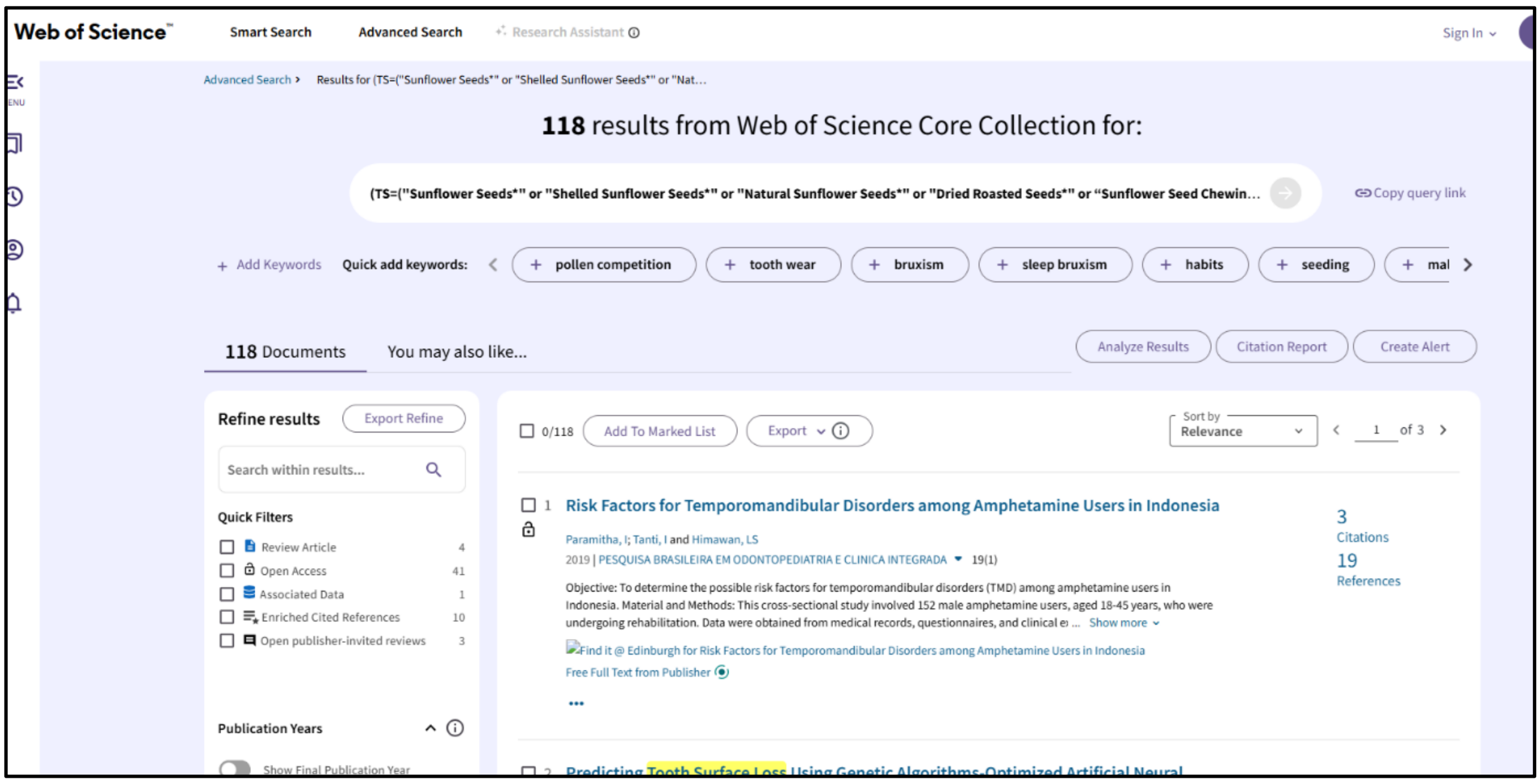

Supplementary Figure S5: Screenshot of search results in Web of Science

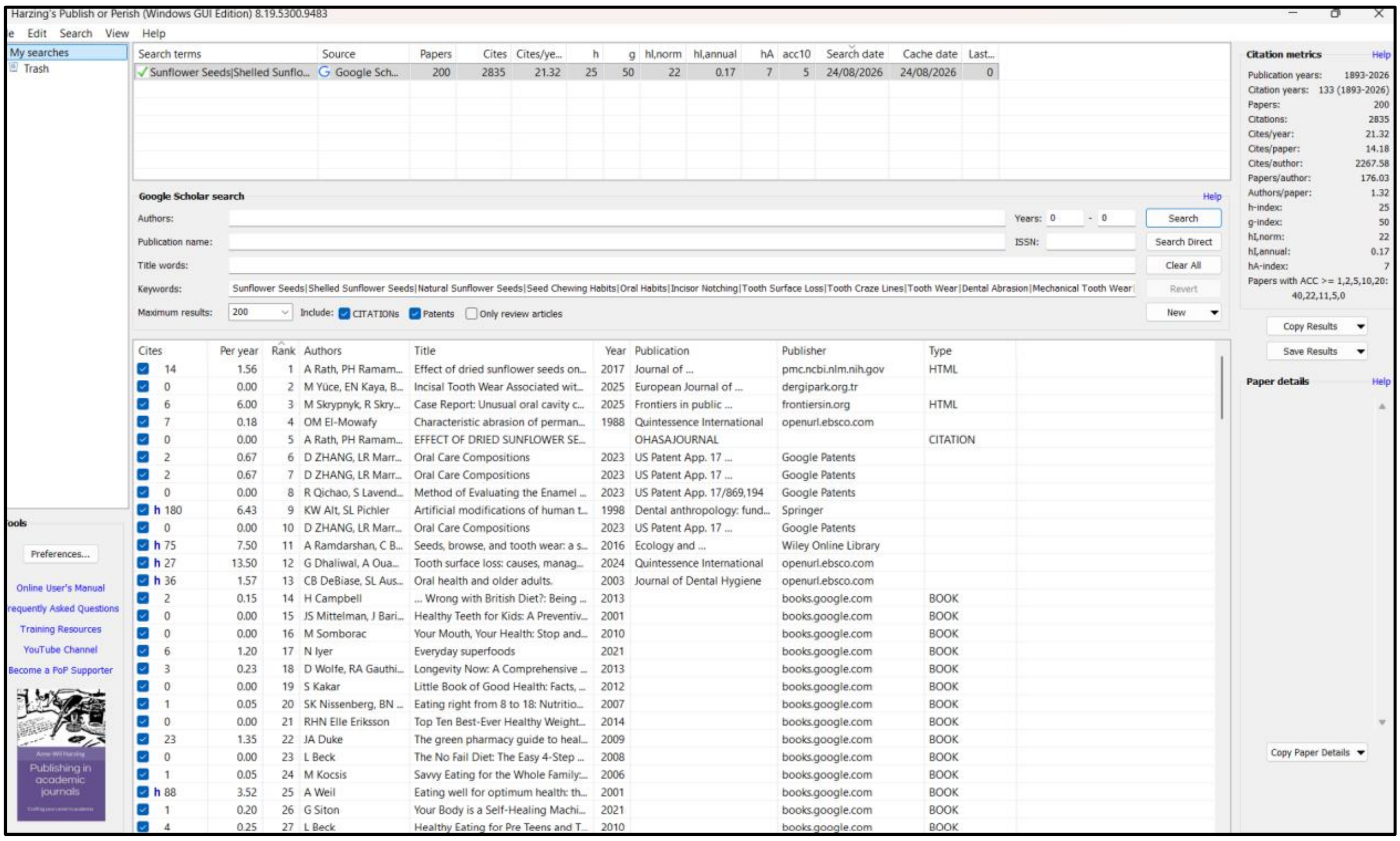

Supplementary Figure S6: Screenshot of search results from Google Scholar obtained using Publish and Perish software (Harzing, A.W. (2007) Publish or Perish, available from https://harzing.com/resources/publish-or-perish.)

Supplementary Table S2: Articles included in the review		
**No**	**Year**	**Articles included in the review**
1	2011	Al-Habahbeh MR, Al-Batayneh OB, Al-Jutaily S, Dar-Odeh NS, Abu-Hammad OA. Effect of dried roasted seeds on tooth surface loss. *Pak Oral Dent J.* 2011;31(2):439-442.
2	2014	Bidra AS. Nonsurgical management of inflammatory periimplant disease caused by food impaction: a clinical report. *J Prosthet Dent.* (2014) 111(2):96–100.
3	2017	Rath A, Ramamurthy PH, Fernandes BA, Sidhu P. Effect of dried sunflower seeds on incisal edge abrasion: A rare case report. *J Conserv Dent. (*2017) 20(2):134–6.
4	2019	Rusu Olaru A, Popescu MR, Dragomir LP, Popescu DM, Arsenie CC, Rauten AM. Identifying the Etiological Factors Involved in the Occurrence of Non-Carious Lesions. *Curr Health Sci J.* (2019) 45(2):227–34.
5	2020	Warreth A, Abuhijleh E, Almaghribi MA, Mahwal G, Ashawish A. Tooth surface loss: A review of literature. *Saudi Dent J.* (2020) 32(2):53–60.
6	2025	Yüce M, Gümrü Tarçın B, Kaya EN, Tarçın B. Incisal Tooth Wear Associated with Prolonged Sunflower Seed Chewing: A Case Report. *Eur J Res Dent.* (2025) 9(3):215–20.
7	2025	Vanishree HS, Tegginamani A. Unusual incisor tooth wear: A diagnostic sign of dietary habits. *Acta Stomatol Naissi.* (2025) 41 (92):3137–3141.
8	2026	Veerabhadrappa SK, Ramachandra SS, Yadav S, Lee XY, Leng LG. Oral Health Implications of Shelled Sunflower Seed Chewing: A Clinical Case Series. *Oral Dis.* (2026) 32(1):251-53.

Supplementary Table S3: Articles excluded after full-text review and reasons for exclusion.		
**No**	**Article details**	**Reasons for exclusion**
**1**	Merchant AT, Pitiphat W, Franz M, Joshipura KJ. Whole-grain and fiber intakes and periodontitis risk in men. Am J Clin Nutr. 2006 Jun;83(6):1395-400.	No exposure to unshelled sunflower seeds.
**2**	Wang X, Cheng C, Ge C, Wang B, Gan YH. Consumption of baked nuts or seeds reduces dental plaque acidogenicity after sucrose challenge. Am J Dent. 2016 Jun;29(3):145-8.	No exposure to unshelled sunflower seeds.
**3**	Zwertvaegher IK, Foqué D, Devarrewaere W, Verboven P, Nuyttens D. Assessment of the abrasion potential of pesticide-treated seeds using the Heubach test. Int J Pest Manag. 2016 Oct 1;62(4):348-359.	No exposure to unshelled sunflower seeds.
**4**	Ramdarshan A, Blondel C, Brunetière N, Francisco A, Gautier D, Surault J, Merceron G. Seeds, browse, and tooth wear: a sheep perspective. Ecol Evol. 2016 Jul 14;6(16):5559-69.	Animal study
**5**	Pini-Prato G, Perrini N, Tardelli M. Periodontal Pocket: An Ecologic Niche Promoting the Germination of a Plant Seed. Int J Periodontics Restorative Dent. 2020 Sep/Oct;40(5):e205-e209.	No exposure to unshelled sunflower seeds
**6**	Manohar R, Ganesh A, Abbyramy N, Abinaya R, Balaji SK, Priya SB. The effect of fennel seeds on pH of saliva - A clinical study. Indian J Dent Res. 2020 Nov-Dec;31(6):921-923.	No exposure to unshelled sunflower seeds
7	Griessl T, Zechel-Gran S, Olejniczak S, Weigel M, Hain T, Domann E. High-resolution taxonomic examination of the oral microbiome after oil pulling with standardized sunflower seed oil and healthy participants: a pilot study. Clin Oral Investig. 2021 May;25(5):2689-2703.	Dental findings not mentioned
8	Patoli I, Kazim M, Jamil SB, Abbas SH. Hypercalcaemia Caused by Sunflower Seeds and Calcium Carbonate Supplements. Eur J Case Rep Intern Med. 2021 Oct 13;8(10):002845.	Dental findings not mentioned
9	An J. Anaphylaxis to Sunflower Seed with Tolerance to Sunflower Oil: A Case Report. Medicina (Kaunas). 2021 Jun 27;57(7):661.	Dental findings not mentioned
10	Baloh CH, Hsu JT. Oral immunotherapy with sunflower seed butter and a review of seed allergy. Allergy Asthma Proc. 2024 Mar 1;45(2):137-140.	Dental findings not mentioned
11	de Alencar Camacho ELC, Carneiro PMA, Loretto SC, de Oliveira RP, Silva AM, Besegato JF, Otárola WGE, de Melo Alencar C. Do polyphenol-rich solutions derived from tropical fruit seeds protect dentin against erosive-abrasive challenges? Clin Oral Investig. 2026 Jun 20;30(7):297.	No exposure to unshelled sunflower seeds
12	Skrypnyk M, Skrypnyk R, Petrushanko T, Skikevych M, Petrushanko V, Skrypnyk I. Case Report: Unusual oral cavity	The reported TSL was associated

	changes associated with methamphetamine abuse. Front Public Health. 2025 Apr 2;13:1473584.	with methamphetamine abuse and could not be specifically attributed to USS consumption.
13	Allareddy V Incidental findings on cone beam computed tomography. Theses and Dissertations, Iowa Research Online, University of Iowa	Mentions about "nut notch" but does not mention the type of seed associated with TSL.
